# Genetic parameters for residual feed intake, methane emissions, and body composition in New Zealand maternal sheep

**DOI:** 10.3389/fgene.2022.911639

**Published:** 2022-08-16

**Authors:** Patricia L. Johnson, Sharon Hickey, Kevin Knowler, Janine Wing, Brooke Bryson, Melanie Hall, Arjan Jonker, Peter H. Janssen, Ken G. Dodds, John C. McEwan, Suzanne J. Rowe

**Affiliations:** ^1^ Invermay Agricultural Centre, AgResearch Ltd., Mosgiel, New Zealand; ^2^ Ruakura Research Centre, AgResearch Ltd., Hamilton, New Zealand; ^3^ Woodlands Research Station, AgResearch Ltd., Woodlands, New Zealand; ^4^ Grasslands Research Centre, AgResearch Ltd., Palmerston North, New Zealand

**Keywords:** residual feed intake, methane, sheep, heritability, body composition

## Abstract

There is simultaneous interest in improving the feed efficiency of ruminant livestock and reducing methane (CH_4_) emissions. The relationship (genetic and phenotypic) between feed efficiency (characterized as residual feed intake: RFI) and greenhouse gases [methane (CH_4_) and carbon dioxide (CO_2_)] traits in New Zealand (NZ) maternal sheep has not previously been investigated, nor has their relationship with detailed estimates of body composition. To investigate these relationships in NZ maternal sheep, a feed intake facility was established at AgResearch Invermay, Mosgiel, NZ in 2015, comprising automated feeders that record individual feeding events. Individual measures of feed intake, feeding behavior (length and duration of eating events), and gas emissions (estimated using portable accumulation chambers) were generated on 986 growing maternal ewe lambs sourced from three pedigree recorded flocks registered in the Sheep Improvement Limited database (www.sil.co.nz). Additional data were generated from a subset of 591 animals for body composition (estimated using ultrasound and computed tomography scanning). The heritability estimates for RFI, CH_4_, and CH_4_/(CH_4_+CO_2_) were 0.42 ± 0.09, 0.32 ± 0.08, and 0.29 ± 0.06, respectively. The heritability estimates for the body composition traits were high for carcass lean and fat traits; for example, the heritability for visceral fat (adjusted for body weight) was 0.93 ± 0.19. The relationship between RFI and CH_4_ emissions was complex, and although less feed eaten will lead to a lowered absolute amount of CH_4_ emitted, there was a negative phenotypic and genetic correlation between RFI and CH_4_/(CH_4_+CO_2_) _of_ −0.13 ± 0.03 and −0.41 ± 0.15, respectively. There were also genetic correlations, that were different from zero, between both RFI and CH_4_ traits with body composition including a negative correlation between the proportion of visceral fat in the body and RFI (−0.52 ± 0.16) and a positive correlation between the proportion of lean in the body and CH_4_ (0.54 ± 0.12). Together the results provide the first accurate estimates of the genetic correlations between RFI, CH_4_ emissions, and the body composition (lean and fat) in sheep. These correlations will need to be accounted for in genetic improvement programs.

## 1 Introduction

There is simultaneous interest in improving the feed efficiency of ruminant livestock and reducing methane (CH_4_) emissions (Archer et al., 2017). At the same time there is an increasing awareness of the importance of moderate body condition, especially moderate body fatness, in conferring positive outcomes in ruminant production systems (Kenyon et al., 2014). As described below it is likely that these traits will share, at least in part, common biological processes, and as such it is important to gain insight into the relationships between these traits.

Feed efficiency is an economically important trait in all production species (Archer et al., 2017). There are many ways in which feed efficiency can be expressed, but one of the most common is residual feed intake (RFI) which was first described by Koch et al. (1963) and is an estimate of whether an animal is consuming more or less energy for its biological outcomes than predicted. The heritability of the trait of RFI in cattle was estimated through a meta-analysis of 39 published articles to be 0.33 ± 0.01 (range of 0.07–0.62) (Berry and Crowley 2013). This large range in estimates is likely due to differences in accuracy of records and length of recording, together with the level of genetic variation in animals measured. There are comparatively few heritability estimates for measures of RFI or alternative measures of feed efficiency in sheep, but such estimates range from 0.11 to 0.49 (Cammack et al., 2005; Fogarty et al., 2006; Paganoni et al., 2017; Tortereau et al., 2020). To date, only preliminary genetic parameter estimates have been published for NZ maternal sheep breeds (Johnson et al., 2018).

Methane (CH_4_) production is closely associated with feed intake, so the link between increased efficiency and CH_4_ emitted is becoming an increasingly important aspect of production and research. Specifically, in New Zealand, farmed ruminants contribute 35% of the country’s greenhouse gas (GHG) emissions (MFE, 2017). Methane expressed in absolute amounts or as a proportion of intake is heritable in sheep as reported by several authors (Pinares-Patiño et al., 2013; Paganoni et al., 2017; Jonker et al., 2018). Methane production is closely associated with feed intake so whether increased efficiency also means less CH_4_ emitted is becoming an increasingly important aspect of production research. Investigating these relationships is critical because as reviewed by Pickering et al. (2013), based on the findings of Blaxter and Claperton (1964) there is a relationship between intake and CH_4_ yield, whereby at lower intakes CH_4_ yield (expressed as g CH_4_/kg DMI) is higher. However, the genetic correlation between RFI and CH_4_ is unknown in sheep. That one exists is, however, plausible given changes in short chain fatty acids ratios are associated with reduced CH_4_ production, specifically reduced acetate relative to propionate (Jonker et al., 2020a) and acetate is the primary source of energy metabolism in adipose tissue (Bergman, 1990).

The relationship between RFI and body composition is also important to consider, as the majority of beef studies have concluded that there is a negative relationship between body composition and feed efficiency, such that more efficient animals are leaner (Hebart et al., 2016), which within maternal production systems is undesirable, and is thought to contribute to delayed onset of puberty and longer calving intervals in cattle (Arthur et al., 2001; Basarab et al., 2011; Donoghue et al., 2011). There are no detailed studies on genetic and phenotypic correlations between CH_4_ and detailed body composition in any species, although there is evidence that animals that had genetically lower CH_4_ yields [expressed as CH_4_/(CH_4_ + CO_2_)] also produced carcasses with proportionately more lean, based on carcass composition prediction from a commercial abattoir grading system (Elmes et al., 2014).

To address the lack of a combined dataset estimating RFI, CH_4_, and body composition on the same maternal sheep, a feed intake facility utilizing custom built automated feeders was established in 2015 at AgResearch Invermay, Mosgiel, NZ (Johnson et al., 2016). The data from the first cohort measured through the facility in 2015 indicated that the level of phenotypic variation in RFI being generated was like that observed in cattle (Johnson et al., 2016) providing confidence in the data being collected. Portable Accumulation Chambers (PAC) designed to measure CH_4_ emissions (Jonker et al., 2018; Jonker et al., 2020b) were already available, as was access to a commercial Computed Tomography (CT) scanning facility for estimating body composition (Jopson et al., 1997).

Here, we describe the use of automated feeders, PAC, and CT scanning to measure 986 young female sheep to investigate genetic and phenotypic correlations between RFI, CH_4_, and body composition.

## 2 Materials and methods

All animal experiments were conducted to meet the guidelines of the 1999 New Zealand Animal Welfare Act and were approved by the AgResearch Grasslands (Palmerston North, NZ) and AgResearch Invermay (Mosgiel, NZ) Animal Ethics committees. Specific approval numbers were AEC13563, AEC13892, and AEC14221.

### 2.1 Animals

Animals used in this study were ewe lambs, with measurements related to this study recorded when the lambs were approximately 9 months of age. Data were collected over 3 years (2015–2017) in cohorts of approximately 200 lambs. In Year 1 (2015) a single cohort (Cohort1) of 200 ewe lambs was measured; in Year 2 (2016) and Year 3 (2017), two cohorts of 200 ewe lambs were measured back-to-back per year (Cohort2a and Cohort2b; Cohort3a and Cohort3b, respectively). The ewe lambs used were of New Zealand maternal genetics sourced from one of three genetically linked flocks: the Beef + Lamb New Zealand Genetics Central Progeny Test (SIL flock number 4640; *n* = 400) which represents a variety of maternal breeds as described by McLean et al. (2006), the AgResearch Research flock (SIL flock number 2638; *n* = 408) (Jonker et al., 2018), which historically had a genetic base of Coopworths, but has been extended to include ewes sired by New Zealand maternal industry sires of different breeds, and the CH_4_ selection lines (SIL flock number 3633; *n* = 192) (Pinares-Patiño et al., 2013). Each sire within these progeny tests was represented in study by five to ten progeny randomly selected from the total available ewe lambs born each year from each resource, except for link sires between years and between progeny tests which had 5–10 progeny in each independent (progeny test or year) resource. All lambs were farmed on the AgResearch Woodlands research farm, near Invercargill, NZ and were re-located to the feed intake facility for measurement by a commercial trucking company, a journey of approximately two and a half hours. Whilst 1,000 animals were measured through the facility, successful datasets were only collected for 986 of them.

### 2.2 Measurements

#### 2.2.1 Feed intake

**FIGURE 1:**
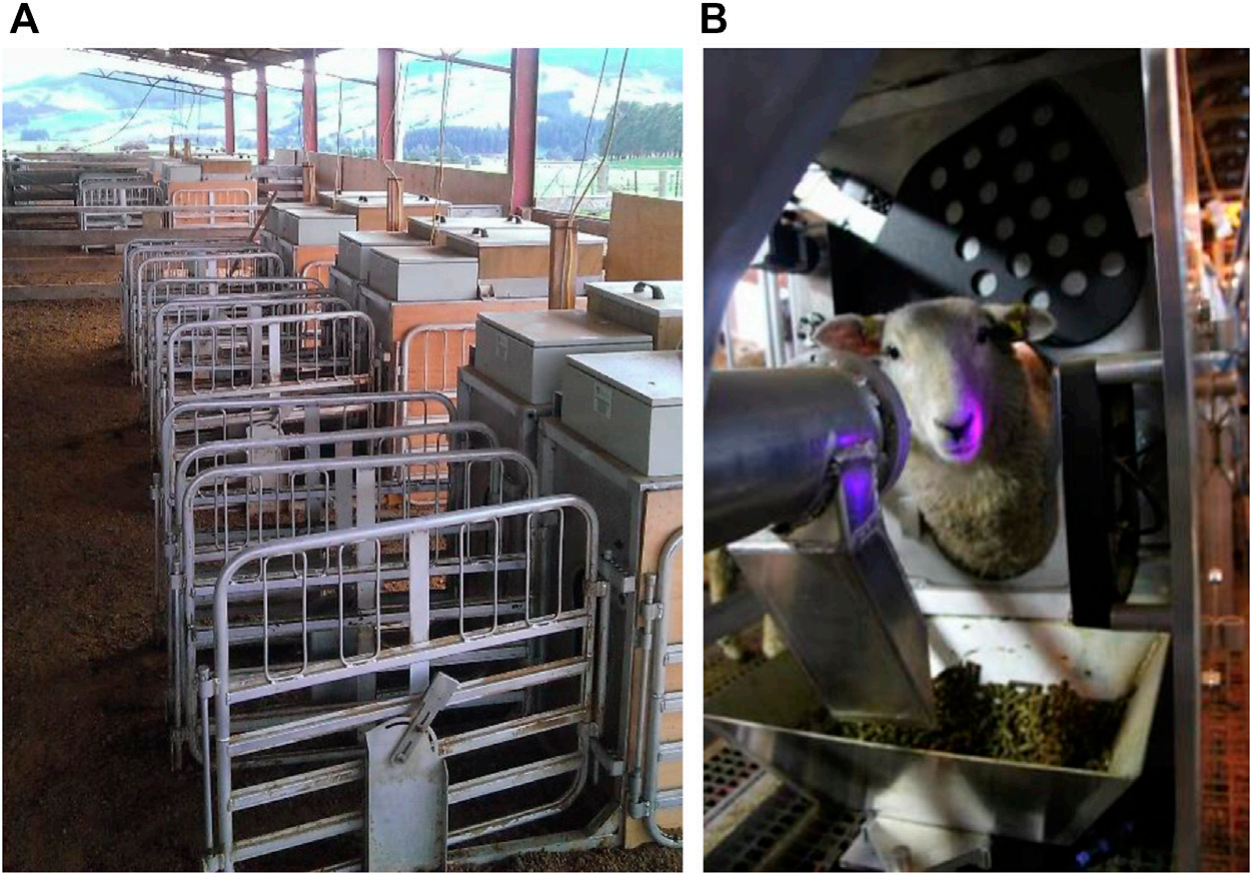
**(A)** Setup of one side of sheep feed intake facility based at AgResearch Invermay, Mosgiel, New Zealand including ten automated feeders; **(B)** internal view of an automated feeder including the metal feed trough fitted on weigh load cells containing the alfalfa pellets and the radio frequency identification (RFID) panel reader as the black panel to the left of the image utilized to read the unique RFID tags fitted to the animals.

Feed intake data were collected using 20 custom made automated feeders designed to allow delivery of pelleted feed. A ratio of 10 animals to each feeder was provided allowing the intake of up to 200 animals to be measured at one time. The feeders were designed by AgResearch Ltd. engineering staff and utilized a metal feed trough fitted on weighing load cells with an automated feed delivery of alfalfa pellets through an auger. Only one animal could access a feeder at a time, by placing its head through a keyhole-shaped opening providing access to a metal feed trough. At all times animals had unrestricted access to the feeders. Unrestricted access to water was also provided through reticulated water troughs. When full, the feed trough contained 2.5 kg of pelleted feed. If the feed available at the end of a feeding event was recorded to be lower than 1 kg, a door would close across the keyhole and the amount of feed would be increased to 2.5 kg. All animals were fitted with radio frequency identification (RFID) ear tags, which were read by panel readers fitted within the feeders. The entry and exit times, together with the weight of feed consumed were recorded in real-time against the animal RFID. An illustration of the feeders is provided in Figure 1.

For the first 2 years (Cohorts 1, 2a, and 2b), the feeders were fitted within an indoor facility, located near AgResearch Invermay, Mosgiel, NZ, consisting of five pens of equal size across a raised floor shed, with four feeders fitted in each pen. Each pen housed a maximum of 40 animals. Individual animals had access to up to four feeders. Due to the sale of the indoor facility, it could no longer be utilized and in the third and final year (Cohorts 3a and 3b), the feeders were re-fitted into a semi-open shed, at AgResearch Invermay, Mosgiel, NZ, divided into 2 pens with 10 feeders each. Each pen could house 100 animals, enabling individual animals to access up to 10 feeders. This facility is illustrated in Figure 1. At all times animals within a pen had access to all feeders within the pen.

The ewe lambs were introduced to alfalfa pellets in the facility over 14 days (sourced from JT Johnson & Sons Ltd., Kapunda, South Australia, Australia; details of the feed used each year are provided in Table 1). Following the introductory 14-day period (introductory period), the main test period was 42 days (test period), with animals housed in the facility for a total of 56 days. The total amount of feed was summed per animal per day to provide an estimate of the animal’s daily feed intake measured as grams of pellets per day. The live weight of the animals was recorded twice weekly in the morning, un-fasted throughout the main test period.

**TABLE 1:** Composition of alfalfa pellets used through feed intake facility across 3 years^a^.

Year	Dry matter content %	Crude protein %	Acid detergent fiber %	Neutral detergent fiber %	Ash %	Fat %	Metabolizable energy (MJ ME)
1	93	14.8	27.0	34.0	9.33	2.3	10.9
2	94	20.0	24.5	22.9	12.8	1.9	11.4
3	90	16.9	31.5	39.0	10.5	1.8	10.8

a Composition data were provided by the pellet manufacturer (JT Johnson and Sons) using an inhouse Foss NIRS DS2500 machine, with data calibrated for hard feed produced by the manufacturer.

#### 2.2.2 Gases

Measures of methane (CH_4_), carbon dioxide (CO_2_), and oxygen (O_2_) were made using PACs as described by Jonker et al. (2018), Jonker et al. (2020b) and similar to those described by Goopy et al. (2011). Briefly, the PACs were made of polycarbonate sheet with an internal volume of 827 L (1.17-m length × 1.15-m height × 0.615-m width). There are 12 chambers aligned as 2 groups of 6 that face each other, however, given that 200 animals were to be assessed, only 10 chambers were used for any single measurement period, resulting in 20 measurement times carried out over two and a half days. Given each chamber is an independent unit, so not utilizing two chambers had no impact on the data collected. Animals were allocated to a time period and chamber using a randomized incomplete block design with allocation to a measurement “lot” of 10 animals. Four groups of animals were removed from feed at a given time, and the first group was off feed for a minimum of 1 hour before they were placed in the individual chambers. Concentrations of CH_4_, CO_2_, and O_2_ in the chambers were determined using an ENVCO Eagle 2 hand-held gas meter (The Environmental Collective Ltd., Auckland, NZ) at the start (0 min), middle (∼30 min), and end (∼60 min) of the PAC measurement period along with the exact time of measurement. Ambient temperature and atmospheric pressure were also recorded.

Gas measurements were made during the third week the animals were in the feed intake test period (5 weeks after entering the facility; round 1) and repeated after 2 weeks with animals completely re-allocated to a time period (lot) and chamber (round 2). This re-allocation was only made for the PAC measurements, with the animals remaining within their RFI pen group whilst in the feed intake facility for the duration of the test period. Successful repeat PAC measurements were made on 986 animals.

#### 2.2.3 Body composition

All lambs in this study were scanned using B-mode ultrasound by a commercial ultrasound assessor before the start and at end of the feed intake test period to measure the depth of subcutaneous fat depth over the *M. longissimus* (C) (Palsson, 1939).

For Cohorts 1, 2a, and 2b, body composition after the feed intake test period (42 days) was also estimated using computed tomography (CT) scanning. CT scanning was limited to 40 animals per day, and so was carried out in the 5 days after completion of the test period, but the animals were still offered access to the alfalfa pellets until they were scanned. Animals were randomly allocated to a day and order of scanning using a randomized incomplete block design. In preparation for scanning, sheep were sedated with Acetylpromazine (Ace10) given by intramuscular injection (1 ml/50 kg body weight). The animals were scanned in a prone position using a custom-built cradle (made of a 400-mm diameter PVC pipe which had been halved with three webbing straps for restraint) with hind legs extended caudally and forelegs under the chest. The animal was scanned using X-ray computed tomography (CT; GE LightSpeed 5.X Pro16 GE Healthcare, Australia) using the procedure described by Jopson et al. (1997) with a random starting position approximately before the 2nd or 3rd cervical vertebrae, through to the distal femur/proximal tibia. Images were collected at 30-mm intervals, with a 450-mm field of view and 5-mm slice thickness. A total of 30–32 images were collected for each animal. The images were initially manually segmented to consist of images that contained the internal organs (visceral components) and their associated fat, or images of the carcass component of the animal. Each of the carcass images was further segmented such that the subcutaneous fat and fat associated with muscles (intermuscular) were separated. The images containing visceral components were further segmented to remove rumen contents. Areas of fat, lean, and bone were calculated in each image using the AUTOCAT program as described by Jopson et al. (1995). The tissues were separated into the three tissue types according to their Hounsfield units (HU). HU value ranges were 40–115, 116–200, and 201–255HU for fat, lean, and bone, respectively. Tissue area (count of pixels) from each image was numerically integrated and multiplied by the distance between images to estimate the tissue volume for the three fat depots, subcutaneous, intramuscular, and visceral fat, as well as total lean, total bone, and non-fat visceral components (Gundersen et al., 1988). Average pixel density was determined by weighting the average density in the individual images by the pixel area in each image. Pixel density was converted to physical density using the relationship shown between HU value and density by Fullerton and Zagzebski (1980). Successful CT data were obtained for 591 animals.

### 2.3 Analysis

#### 2.3.1 Residual feed intake

Live weight data collected across the feed intake test period (42 days) was modeled to determine the live weight change profile of the animals from which the growth rate of the animals and the metabolic mid-weight (Day 21) were calculated. Two approaches were considered for modeling the data; 1) a linear regression model fitted through the data points where x = day of measurement and y = live weight as described in Johnson et al. (2015), and 2) through using interpolation where the weights on days between measurements were calculated by estimating the daily gain between two consecutive live weights. Both methods produced highly correlated (>0.95) results (data not presented). Results from the interpolation method are presented below.

The average intake of the animals was calculated as the mean of the 42 daily intakes of the animals across the test period, measured in grams of pellets per day. This was converted to energy intake using the information on the feed as described in Table 1, where

$$Energy\;Intake\;\left(MJME\right)=\frac{\left(Feed\;Intake*Dry\;Matter\;Content\right)}{Metabolisable\;Energy\;Content}$$

The trait of RFI was calculated based on the model of Koch et al. (1963).

 y = β0 + β1∗MMWT + β2∗ADG + Flock + Cohort + Pen + ε

where y is the measured energy intake (calculated prior using the MIXED procedure in SAS, fitting day as a repeated measure), β0 = intercept, β1 and β2 are estimated coefficients, MMWT = metabolic mid-weight (weight at Day21^0.75^), ADG = average daily gain as estimated from using the bi-weekly liveweight measurements and the day of measurement (with the first measurement made on day 0), Flock = Flock A, B or C, Cohort = Cohort 1, 2a, 2b, 3a or 3b, Pen=(Cohort1, Cohort2a, Cohort 2b:A-E; Cohort3a, Cohort3b:A-B), and *ε* = the residual which is taken as the trait of RFI.

Additional feeding behavior traits were calculated from the feed intake data including the average number of feeding events an individual animal undertook per day, the average duration of each feeding event, the average amount consumed per feeding event, and the rate of feeding during each event (intake/duration).

#### 2.3.2 Gases

The raw gas data were converted to appropriate measures as described by Jonker et al. (2018) to result in the traits being expressed as moles and grams of CH_4_ and CO_2_ produced per day. The gas traits were scaled to adjust for the variance within the contemporary PAC lot (group of 10 animals measured together) due to varying waiting times after removal from pasture. The ratio of actual value/lot mean was multiplied by the overall trait mean. Absolute values of CH_4_ and CO_2_ are reported together with their sum and the derived trait of CH_4_/(CH_4_+ CO_2_) (mol/mol). This trait is a description of CH_4_ yield with CH_4_+ CO_2_ considered as a proxy of intake (Jonker et al., 2018) but it is explicitly reported as the formula in this article as the extended literature reports many different versions of CH_4_ yield (different denominators).

#### 2.3.3 Body composition

Several body composition traits were derived from the CT images. Adipose traits generated were the absolute amounts of subcutaneous fat, visceral fat, and intermuscular fat. Carcass fat as would be observed on a carcass post-slaughter was calculated as the sum of subcutaneous and intermuscular fat, with total fat calculated as the sum of subcutaneous, visceral, and intermuscular fat. Carcass lean and bone were calculated as the sum of all estimates of lean and bone, respectively. Carcass weight was estimated as the sum of carcass fat, lean, and bone. Computed tomography weight was calculated as the sum of carcass weight, visceral fat, and non-fat visceral components and is the equivalent of a fasted body weight (no gut fill). Dressing-out-percent was calculated as the ratio of carcass weight to computed tomography weight.

#### 2.3.4 Genetic parameters

Variance components were estimated using restricted maximum likelihood (REML) procedures fitting an animal model in ASReml 3.0 (Gilmour et al., 2009). Pedigree records were obtained from Sheep Improvement Limited for all three birth flocks of animals, born from 1990 to 2016 and a pedigree file was constructed. Univariate models were used to estimate heritabilities ADG, MMWT, RFI, the carcass composition traits (ultrasound and CT), feeding behavior traits, and gas traits. Final fixed effects that were significant (*p* < 0.05) are summarized in Table 2. Additionally, for the gas traits, a repeated measures model was fitted to account for the two measurements that were made on each individual, with round (1 or 2) fitted as a repeated measure. Dam was also fitted as a random effect in all models to allow estimation of both direct and maternal heritability. Bivariate models were used to estimate the genetic and phenotypic correlations between RFI and the gas traits and between these and all other traits. Genetic parameter estimates were considered to be different from zero when they were more than two times the standard error estimates (Rowland et al., 2019).

**TABLE 2:** Significant effects (*p* < 0.05) in the variance component analysis, fitting fixed effects of contemporary group (cg:birth year-birth flock), cohort-pen, and birth-rearing rank (brr), with covariates of age of dam (aod), quadratic of age of dam (aod^2^), birthday deviation (bdev), metabolic mid-weight (MMWT) for body composition traits, and empty body weight (CTWT) for the computed tomography traits. PAC gas traits are scaled by PAC contemporary group (measurement date—PAC lot).

Trait	Significant effects (*p* < 0.05)
Traits in residual feed intake model	
Intake	cg, cohort.pen,brr, aod^2^
Metabolic mid-weight	cg, brr, aod, aod^2^, bdev
Growth rate	cg, cohort.pen, brr
Feeding behavior	
Average feeding time per feeding event	cg, cohort.pen, brr
Average intake per feeding event	cg, cohort.pen, brr
Average number of daily feeding events	cg, cohort.pen
Average feeding rate per feeding event	cg, cohort.pen, brr
Ultrasound assessed body composition	
Starting C^a^	cg, cohort.pen, MMW
Final C^a^	cg, cohort.pen, MMW
Change in C^a^	cg, cohort.pen, MMW
Computed tomography assessed body comp	
Visceral fat	cg, CTWT
Subcutaneous fat	cg, CTWT
Intermuscular fat	cg, CTWT
Carcass lean	cg, brr
Carcass fat	cg, CTWT
Total fat	cg, CTWT
Total bone	cg, CTWT
Non-fat visceral components	cg, CTWT
Fat:lean	cg, CTWT
Carcass weight^b^	cg
Dressing-out-percent^c^	cg, CTWT
Portable accumulation chamber measurements	
Body weight	cg, cohort, brr, aod, aod^2^, bdev
CH_4_	cg, brr, aod^2^
CO_2_	cohort, brr
CH_4_ + CO_2_	cohort, brr
CH_4_/(CH_4_ + CO_2_)	cg, cohort, aod^2^

a C is the measurement of subcutaneous fat depth over the *M. longissimus lumborum* using ultrasound.

b Estimated as the sum of weights of carcass components.

c Ratio of carcass weight to live weight.

## 3 Results

Full feed intake and growth data from which the trait of RFI could be calculated were available for 986 growing ewe lambs, measured when they were approximately 9–12 months of age. Summary statistics for traits measured are provided in Table 3. The average mid-trial live weight for the animals across cohorts was 57.2 kg which equates to a MMWT of 20.8 ± 2.0 kg [MMWT is the trait reported in many RFI studies (Archer et al., 1997)]. The lambs grew on average 0.35 ± 0.067 kg per day during the duration of the trial period. For the five individual cohorts, the R^2^ of the model fitted to the energy intake data, from which the trait of RFI was estimated, was between 0.69 and 0.78.

**TABLE 3:** Summary statistics (mean, s.d), and heritability (h^2^) estimates (±s.e.) (direct and maternal) for residual feed intake and associated production, and feed behavior traits together with body composition traits measured at the time of feed intake data being collected. Feed intake, production, and ultrasound results are based on data from 986 animals, computed tomography scanning trait results are based on data from a subset of 591 animals.

Trait	Mean	s.d.	Direct	Maternal
			h^2^ ± s.e.	h^2^ ± s.e.
Residual feed intake (MJ/day)	−0.00094	1.328	0.42 ± 0.09	
Traits in residual feed intake model				
Feed intake (MJ/day)	25.6	4.57	0.35 ± 0.10	0.08 ± 0.06
Mid-trial metabolic live weight (kg)	20.8	2.0	0.44 ± 0.11	0.16 ± 0.07
Growth rate (kg/day)	0.35	0.067	0.42 ± 0.10	0.02 ± 0.06
Feeding behavior				
Average feeding time per feeding event- (sec)	547.7	179.5	0.58 ± 0.11	0.04 ± 0.06
Average intake per feeding event (g)	203.8	69.92	0.47 ± 0.09	
Average number of daily feeding events	13.67	4.427	0.44 ± 0.09	
Average feeding rate per feeding event	0.3846	0.105	0.29 ± 0.10	0.08 ± 0.06
Ultrasound assessed body composition				
Starting C (mm)^a^	3.85	1.156	0.39 ± 0.11	0.06 ± 0.06
Final C (mm)^a^	4.58	1.37	0.57 ± 0.09	
Change C (mm)^a^	0.74	1.097	0.15 ± 0.07	
Computed tomography assessed body comp				
Visceral fat (kg)	5.13	1.33	0.93 ± 0.19	0.01 ± 0.09
Subcutaneous fat (kg)	5.51	1.76	0.59 ± 0.17	0.12 ± 0.10
Intermuscular fat (kg)	2.29	0.62	0.72 ± 0.18	0.10 ± 0.10
Carcass lean (kg)	19.10	2.38	0.81 ± 0.18	0.08 ± 0.09
Carcass fat (kg)	7.80	2.33	0.68 ± 0.18	0.10 ± 0.10
Total fat (kg)	12.93	3.56	0.71 ± 0.18	0.12 ± 0.10
Total bone (kg)	4.36	0.47	0.28 ± 0.12	0.15 ± 0.10
Non-fat visceral components (kg)	10.76	1.57	0.56 ± 0.13	
Fat:lean	0.67	0.15	0.82 ± 0.19	0.08 ± 0.10
Carcass weight (kg)^b^	31.26	4.52	0.77 ± 0.14	
Dressing-out-percent (%)^c^	48.81	2.16	0.82 ± 0.14	

a C is the measurement of subcutaneous fat depth over the *M. longissimus lumborum* using ultrasound.

b Estimated as the sum of weights of carcass components.

c Ratio of carcass weight to live weight.

The heritability estimates for individual traits measured through the feed intake facility and body composition traits, together with summary details for the traits, are in Table 3. Of the feed intake-related traits, average time per feeding event had the highest heritability (0.58 ± 0.11) and average daily feed intake the lowest (0.35 ± 0.10). Among the body composition traits, the amount of visceral fat had the highest heritability (0.93 ± 0.19) and the change in fat depth C as measured using ultrasound had the lowest heritability (0.15 ± 0.07).

The heritability estimates and the repeatability for the gas traits, together with summary details for the traits are provided in Table 4. The gas traits with the highest heritability were CH_4_ and CO_2_ (0.32 ± 0.08) and the lowest heritability was for CH_4_/(CO_2_ + CH_4_) (0.29 ± 0.06). The repeatability of the gas traits was highest for CO_2_ and CO_2_ + CH_4_ (0.57 ± 0.02).

**TABLE 4:** Summary statistics (mean, s.d), heritability (h^2^) estimates (±s.e.) (direct and maternal), and 14-day repeatability estimates (±s.e.) for body weight (BW) and methane (CH_4_), carbon dioxide (CO_2_), and oxygen (O_2_) measured using a portable accumulation chamber. Results are based on data from 968 animals.

	Mean	s.d.	Direct h^2^ ±s.e.	Maternal h^2^ ± s.e.	Repeat. ± s.e.14 Day
BW (kg)	57.6	8.6	0.43 ± 0.11	0.15 ± 0.07	0.95 ± 0.003
CH_4_ g/day	17.2	3.51	0.32 ± 0.08	0.01 ± 0.04	0.31 ± 0.03
CO_2_ g/day	1,248	235	0.32 ± 0.08	0.04 ± 0.05	0.57 ± 0.02
CH_4_ + CO_2_ (mol)	29.4	5.44	0.31 ± 0.08	0.04 ± 0.05	0.57 ± 0.02
CH_4_/(CH_4_ + CO_2_) (mol/mol)	0.037	0.0070	0.29 ± 0.06		0.32 ± 0.03

The genetic and phenotypic correlations between RFI and the other feed intake and body composition traits are in Table 5. The highest phenotypic between RFI and the feeding behavior traits was with average intake per feeding event (0.16 ± 0.03); none of the genetic correlations were different from zero. The highest phenotypic and genetic correlations between RFI and the body composition traits were for non-fat visceral components (0.33 ± 0.04 and 0.64 ± 0.13). In general, fat traits were negatively genetically correlated with RFI and positively correlated with non-fat visceral components.

**TABLE 5:** Genetic (r_g_) and phenotypic (r_p_) correlations (±s.e.) between the trait of residual feed intake and other production, feeding behavior, and body composition traits estimated on New Zealand maternal sheep. Feed intake, production, and ultrasound results are based on data from 986 animals, with results including CT scanning based on data from a subset of 591 animals.

	Residual feed intake	
	r_g_	r_p_
Traits in residual feed intake model		
Feed intake (MJ/day)	0.41 ± 0.14	0.54 ± 0.03
Mid-trial metabolic live weight (kg)	−0.23 ± 0.17	0.02 ± 0.04
Growth rate (kg/day)	−0.09 ± 0.17	−0.01 ± 0.04
Feeding behavior		
Average feeding time per feeding event	0.17 ± 0.14	0.05 ± 0.04
Average intake per feeding event	0.06 ± 0.16	0.16 ± 0.03
Average number of daily feeding events	0.13 ± 0.16	0.06 ± 0.04
Average feeding rate per feeding event	−0.25 ± 0.19	0.11 ± 0.03
Ultrasound assessed body composition		
Starting C (mm)^a^	−0.15 ± 0.17	−0.20 ± 0.03
Final C (mm)^a^	−0.14 ± 0.15	0.004 ± 0.04
Change C (mm)^a^	−0.04 ± 0.24	0.20 ± 0.03
Computed tomography assessed body comp		
Visceral fat (kg)	−0.52 ± 0.16	−0.11 ± 0.05
Subcutaneous fat (kg)	−0.33 ± 0.19	0.08 ± 0.05
Intermuscular fat (kg)	−0.30 ± 0.18	−0.05 ± 0.05
Carcass lean (kg)	0.06 ± 0.18	−0.06 ± 0.05
Carcass fat (kg)	−0.38 ± 0.19	0.04 ± 0.05
Total fat (kg)	−0.58 ± 0.18	−0.03 ± 0.05
Total bone (kg)	0.48 ± 0.25	0.01 ± 0.05
Non-fat visceral components (kg)	0.64 ± 0.13	0.33 ± 0.04
Fat:lean	−0.41 ± 0.18	0.07 ± 0.05
Carcass weight (kg)^b^	−0.18 ± 0.17	−0.26 ± 0.05
Dressing-out-percent (%)^c^	−0.08 ± 0.18	−0.20 ± 0.05

a C is the measurement of subcutaneous fat depth over the *M. longissimus lumborum* using ultrasound.

b Estimated as the sum of weights of carcass components.

c Ratio of carcass weight to live weight.

The genetic and phenotypic correlations between the gas traits and the feed intake and body composition traits are provided in Table 6. Several correlations were different from zero. There was a negative phenotypic and genetic correlation between CH_4_/(CO_2_ + CH_4_) and RFI (−0.13 ± 0.03 and −0.41 ± 0.15, respectively). Phenotypic and genetic correlations were observed between CO_2_ and daily feed intake and mid-trial live weight (phenotypic correlations 0.45–0.47 with standard errors of 0.2 and genetic correlations of 0.59–0.62 with standard errors of 0.13–0.14) Positive phenotypic and genetic correlations, that were different from zero, were reported between CH_4_ and feeding behavior traits of average intake per feeding event and average feeding rate (phenotypic correlations greater than 0.23 with standard errors of 0.03 and genetic correlations greater than 0.41 with standard errors of 0.13–0.16). There were phenotypic and genetic correlations, that were different from zero, between absolute CH_4_ and carcass lean (0.28 ± 0.03 and 0.54 ± 0.12, respectively) and non-fat visceral components (0.12 ± 0.04 and 0.36 ± 0.17, respectively) estimated from CT scanning. Several other correlations, that were different from zero, between CO_2_ and body composition traits were also found, with strong negative phenotypic genetic correlations between CO_2_ and fat traits, and conversely strong positive correlations with lean traits.

**TABLE 6:** Genetic (r_g_) and phenotypic (r_p_) correlations (±s.e.) between PAC gas production traits (estimated from indoor feeding) and residual feed, feeding behavior, and body composition traits estimated on New Zealand maternal sheep. Results are based on data from 968 animals for which feed intake and PAC data were available.

	CH_4_ g/d		CO_2_ g/d		CH_4_+CO_2_(mol)		CH_4_/(CH_4_+CO_2_)	
	r_g_	r_p_	r_g_	r_p_	r_g_	r_p_	r_g_	r_p_
Residual feed intake (MJ/day)	−0.28 ± 0.16	0.03 ± 0.03	0.05 ± 0.17	0.17 ± 0.03	0.04 ± 0.17	0.17 ± 0.03	−0.41 ± 0.15	−0.13 ± 0.03
Traits in residual feed intake model								
Feed intake (MJ/day)	0.33 ± 0.17	0.36 ± 0.02	0.59 ± 0.14	0.47 ± 0.02	0.60 ± 0.14	0.48 ± 0.02	−0.24 ± 0.09	−0.08 ± 0.03
Mid-trial metabolic live weight (kg)	0.68 ± 0.11	0.41 ± 0.02	0.62 ± 0.13	0.45 ± 0.02	0.73 ± 0.08	0.46 ± 0.02	−0.06 ± 0.08	−0.02 ± 0.03
Growth rate (kg/day)	0.34 ± 0.10	0.27 ± 0.03	0.06 ± 0.19	0.21 ± 0.03	0.07 ± 0.19	0.22 ± 0.03	0.10 ± 0.16	0.07 ± 0.03
Feeding behavior								
Average feeding time per feeding event	0.04 ± 0.16	0.08 ± 0.03	0.001 ± 0.17	0.04 ± 0.03	0.004 ± 0.17	0.04 ± 0.03	0.02 ± 0.15	0.04 ± 0.03
Average intake per feeding event	0.41 ± 0.13	0.26 ± 0.03	0.22 ± 0.16	0.18 ± 0.03	0.24 ± 0.16	0.19 ± 0.03	0.21 ± 0.08	0.08 ± 0.03
Average number of daily feeding events	−0.28 ± 0.08	−0.10 ± 0.03	0.02 ± 0.08	0.01 ± 0.03	0.01 ± 0.08	0.002 ± 0.03	−0.38 ± 0.15	−0.10 ± 0.03
Average feeding rate per feeding event	0.55 ± 0.16	0.23 ± 0.03	0.25 ± 0.21	0.16 ± 0.03	0.27 ± 0.21	0.17 ± 0.03	0.27 ± 0.11	0.07 ± 0.03
Ultrasound assessed body composition								
Starting C (mm)^a^	−0.001 ± 0.13	−0.02 ± 0.03	−0.35 ± 0.14	−0.12 ± 0.03	−0.35 ± 0.14	−0.12 ± 0.03	0.27 ± 0.10	0.08 ± 0.03
Final C (mm)^a^	−0.04 ± 0.15	−0.01 ± 0.03	−0.33 ± 0.15	−0.03 ± 0.03	−0.32 ± 0.15	−0.03 ± 0.03	0.06 ± 0.08	0.02 ± 0.03
Change C (mm)^a^	−0.27 ± 0.23	−0.002 ± 0.03	−0.17 ± 0.25	0.09 ± 0.03	−0.17 ± 0.25	0.09 ± 0.03	−0.26 ± 0.13	−0.06 ± 0.03
Computed tomography assessed body comp								
Visceral fat (kg)	−0.13 ± 0.16	−0.05 ± 0.04	−0.51 ± 0.14	−0.16 ± 0.04	−0.51 ± 0.14	−0.16 ± 0.04	0.31 ± 0.15	0.07 ± 0.04
Subcutaneous fat (kg)	−0.10 ± 0.19	0.03 ± 0.04	−0.42 ± 0.16	−0.14 ± 0.04	−0.44 ± 0.17	−0.14 ± 0.04	0.19 ± 0.17	0.11 ± 0.04
Intermuscular fat (kg)	−0.10 ± 0.18	0.04 ± 0.04	−0.43 ± 0.15	−0.21 ± 0.04	−0.43 ± 0.15	−0.21 ± 0.04	0.24 ± 0.17	0.09 ± 0.04
Carcass lean (kg)	0.54 ± 0.12	0.28 ± 0.03	0.65 ± 0.11	0.41 ± 0.03	0.66 ± 0.11	0.41 ± 0.03	−0.03 ± 0.17	−0.05 ± 0.04
Carcass fat (kg)	−0.11 ± 0.18	0.01 ± 0.04	−0.46 ± 0.16	−0.17 ± 0.04	−0.46 ± 0.16	−0.17 ± 0.04	0.24 ± 0.18	0.12 ± 0.04
Total fat (kg)	−0.13 ± 0.18	−0.01 ± 0.04	−0.57 ± 0.15	−0.19 ± 0.04	−0.56 ± 0.15	−0.19 ± 0.04	0.33 ± 0.17	0.11 ± 0.04
Total bone (kg)	−0.33 ± 0.25	−0.07 ± 0.04	0.54 ± 0.23	0.08 ± 0.04	0.52 ± 0.23	0.07 ± 0.04	−0.72 ± 0.26	−0.09 ± 0.03
Non-fat visceral components (kg)	0.36 ± 0.17	0.12 ± 0.04	0.37 ± 0.17	0.20 ± 0.04	0.39 ± 0.17	0.21 ± 0.04	−0.03 ± 0.18	−0.03 ± 0.04
Fat:lean	−0.12 ± 0.17	0.01 ± 0.04	−0.51 ± 0.14	−0.15 ± 0.04	−0.51 ± 0.14	−0.15 ± 0.04	0.30 ± 0.16	0.10 ± 0.04
Carcass weight (kg)^b^	−0.22 ± 0.16	−0.08 ± 0.04	0.15 ± 0.17	−0.06 ± 0.04	0.13 ± 0.17	−0.06 ± 0.04	−0.28 ± 0.16	−0.04 ± 0.04
Dressing-out-percent (%)^c^	−0.24 ± 0.16	−0.20 ± 0.04	0.23 ± 0.17	−0.08 ± 0.04	0.21 ± 0.17	−0.09 ± 0.04	−0.33 ± 0.15	−0.11 ± 0.04

a C is the measurement of subcutaneous fat depth over the *M. longissimus lumborum* using ultrasound.

b Estimated as the sum of weights of carcass components.

c Ratio of carcass weight to live weight.

## 4 Discussion

### 4.1 Feed intake traits

When this program of work commenced, there was limited literature relating to feed intake in sheep. To calculate the base trait of residual feed intake as first described by Koch et al. (1963), three key pieces of data were required: the intake of individual animals, their liveweight and their growth rate. In cattle, it has been determined that accurate estimates of intake could be calculated after a 35-day test period, but that to accurately estimate growth rate and therefore RFI a 70-day test period was required (Archer et al., 1997). Preliminary research (Johnson et al., 2015) supported the work of Cockrum et al. (2013) that in sheep, a test measurement period of 42 days was sufficient to enable accurate estimation of live weight gain, which was further enhanced through bi-weekly measurements of live weight being made. Subsequent analysis of data associated with Johnson et al. (2015) confirmed that accurate (stabilized) intake data in sheep is available after only a 21–28-day test period (Johnson et al., 2017) and as such the 42-day test period was appropriate for use in sheep. Several other studies on sheep concurrently undertaken by other research groups have similarly employed the 42-day test period (Paganoni et al., 2017; Muir et al., 2018).

The phenotypic variation in RFI was explored by Johnson et al. (2015)using data from Cohort1, where it was demonstrated that animals classified as RFI). This difference was consistent with the results of the sheep studies of Redden et al. (2013) and Cockrum et al. (2013) and a dairy heifer trial of Williams et al. (2011) who observed differences of 17%, 30%, and 20%, respectively, between efficiency group extremes. The size of the heritability estimates for the feed intake-related traits was very consistent with an earlier report based on data from the first three cohorts measured through the facility (Johnson et al., 2018). The size of the RFI heritability estimate is within the range reported for sheep (Cammack et al., 2005; Fogarty et al., 2006; Paganoni et al., 2017; Tortereau et al., 2020) and the cattle range reviewed by Berry and Crowley (2013).

The heritability estimates for the feeding behavior traits were all moderate to high. Between animal variation has been observed in feeding behavior traits by several authors (Cammack et al., 2005; Muir et al., 2018) although the level of genetic variation observed in this study is considerably greater than that observed by Cammack et al. (2005).

### 4.2 Body composition traits

Adipose tissue reserves are a critical component of maternal ewes and are phenotypically and genetically associated with improved maternal production, although the size of the effects are sometimes small (Lambe et al., 2005; Walkom et al., 2015). The most common method of measuring fatness in live sheep is the use of ultrasound to predict the amount of fat over the *M. longissimus* muscle. Whilst this measurement is related to total body composition (Young et al., 1996) and is useful as a low-cost measurement, it is not able to fully predict fat deposition across the different depots. The only options, therefore, for accurately estimating full body composition are through slaughter and dissection, or through using CT scanning. The importance of understanding variation in body fat distribution was highlighted by Lambe et al. (2003) who observed differences in how labile different fat depots were, concluding that visceral fat was the most labile, followed by subcutaneous fat with intermuscular fat the least labile. Thus, there may be advantages in selection for ewes that lay down more of their fat internally, which would also suit prime lamb production systems where excess carcass fat is not desired.

Given the difficulty in generating full body composition data, there are no genetic parameter estimates for full-body composition estimates in sheep. Evidence of between breed variation exists, with McClelland and Russel (1972) demonstrating Finnish Landrace ewes lay down more visceral fat compared with Scottish Blackface ewes. In cattle, the situation is even more limited as there is no option for CT scanning due to the size of the animals, but between cattle types variation in body composition has been estimated through dissection. Wright and Russel (1984) reported that dairy breeds accumulate relatively more fat in visceral adipose depots and less in subcutaneous fat when compared with beef breeds. The results in our study strongly support that fat deposits are under strong genetic control. The standard errors associated with these estimates are higher than those for the RFI and PAC traits due to fewer animals being measured for these traits.

### 4.3 Gas traits

The units of measurement for gas traits estimated from either respiration chambers and/or portable accumulation chambers vary between studies, making their direct comparison difficult. The most comparable literature for this study is that of Jonker et al. (2018) which used the same PAC used in this current Study. The animals were measured at different ages (younger or older) and were measured whilst grazing pasture, although in that study some animals were also measured through respiration chambers using similar alfalfa-based pellets. The absolute emissions of CH_4_ reported in this study are lower than those reported for the respiration chamber measurements of Jonker et al. (2018) but higher than their PAC pasture estimates. The moderate repeatability estimates for the two PAC measures which were approximately 2 weeks apart are consistent with repeatability estimates for consecutive PAC measures (Robinson et al., 2014; Jonker et al., 2018). Although *ad libitum* feed was offered before both time points in the current study, given the spot nature of the measurements (only 1 hour), variation in feed intake before entry into the facility and potential diurnal variation in behavior (even though group was adjusted for in the PAC calculations) may contribute to the repeatability being reduced to that observed from respiration chamber measurements (Jonker et al., 2018).

The heritability estimates for all gas traits are more comparable to those estimated in the respiration chambers by (Jonker et al., 2018) than their equivalent PAC estimates off pasture, and are higher than the estimates of Robinson et al. (2014) and Paganoni et al. (2017) who used PAC to generate data on CH_4_ and CO_2_ from Merino derived sheep fed a pelleted feed and Goopy et al. (2016) from maternal sheep fed an alfalfa sward.

### 4.4 Correlations between feed intake traits and gas traits

As anticipated increasing feed intake was associated with increased absolute emissions. The relationship between feed intake and CH_4_ as described by Blaxter and Claperton (1964) was observed whereby at lower intakes CH_4_ yield is higher. The phenotypic correlation was low, but different from zero, with the genetic correlation higher. Similar results were observed by Jonker et al. (2018) for measurements made using PACs where CH_4_ + CO_2_ was used as a proxy for feed intake. Although unfavorable, the genetic correlations between RFI and CH_4_ are only moderate, suggesting that a multi-trait selection index could make progress in reducing CH_4_ and improving RFI simultaneously.

There is evidence that low CH_4_ sheep have a trend of smaller rumens than their high emitting counterparts (Bain et al., 2014; Elmes et al., 2014; Goopy et al., 2014). The low CH_4_/(CH_4_+CO_2_) sheep measured in this current study showed a trend of smaller, more frequent meals which is consistent with these observations.

It has been postulated that total gas emissions might be used as an indication of metabolic rate and a predictor of feed intake. Table 6 shows moderate to high positive correlations between total gas (CH_4_ + CO_2_) and feed intake. Given that the gas measures were only taken for 1 hour at two time points and are being used to predict feed intake over 42 days, the results suggest that the use of CO_2_ as a low-cost predictor of feed intake warrants further investigation.

### 4.5 Correlations between residual feed intake and body composition traits

The strongest correlation observed between RFI, and the body composition traits measured in this study was a genetic correlation between visceral fat as assessed at the end of the test period and RFI, a correlation never reported in the literature previously because of difficulties in estimating the trait (can only be assessed using CT scanning or carcass dissection). Other results in this study are not consistent with cattle literature, which is based on ultrasound data, in which it is generally concluded that more efficient animals are leaner (Herd et al., 2019). In contrast, the results from this study suggest alternative relationships. All fat traits were adjusted for weight, and as such relate to the composition (relative level of fat at a set weight) of the animal. It should be noted that the CT measurements were taken at the end of the test period, and therefore most closely correspond to the C measurement made at the end of the test period. At the phenotypic level, change in C fat depth across the test period was positively associated with RFI indicating that animals that laid down more fat ate more. This phenotypic relationship has been described in physiological studies in sheep (Ball et al., 1997) based on CT data but has not previously been reported in cattle studies in part given the complexity of getting comparable body composition data. However, independent of fat laid down during the trial period, animals that were initially fatter as assessed by C were more efficient, a relationship observed at the phenotypic level. This is consistent with physiological studies that have shown that although it is more energetically expensive to lay down (deposit) body fat, once deposited the maintenance energy requirements of lean tissue are higher (Ball et al., 1997). There is evidence that different fat depots have different physiological attributes (Berg and Butterfield, 1976; Wright and Russel, 1984) and potentially, therefore, have different relationships with feed intake. Specifically, visceral fat is more labile than other fat depots and therefore more metabolically active than other depots (Lambe et al., 2005).

### 4.6 Correlations between gas and body composition traits

All fat traits were adjusted for weight, and as such relate to the composition (relative level of fat at a set weight) of the animal. There is evidence of a positive correlation between carcass fat traits and CH_4_/(CH_4_ + CO_2_) which is consistent with the finding of Elmes et al. (2014) who investigated differences in carcass composition between CH_4_ yield selection lines and reported lambs from the low CH_4_ yield line to be leaner than those from the high CH_4_ line. In this current study the genetic correlations were only low, but still different from zero, for starting C and visceral fat. There were strong negative correlations between CO_2_ and the fat traits and conversely positive with carcass lean, bone, and non-fat visceral components. Carbon dioxide is produced as a by-product of tissue metabolism. A biological interpretation of this relationship is that fat is metabolically inert in a growing animal (Ball et al., 1997). Hence, more body fat results in less CO_2_ being produced per unit Weight. Conversely, lean tissue is more metabolically active and as a result more body lean results in more CO_2_ being produced.

Rumen size was not explicitly estimated from the CT images as it was by Bain et al. (2014), and so a relationship between rumen size and CH_4_ cannot be inferred from this dataset but could be estimated through further processing of the CT images at a future date when resources allow.

### 4.7 Overall considerations

This study has combined a number of difficult and/or expensive to measure traits into one study with the same animals being directly measured for all traits reported in the study (with the exception of a lower number that have CT estimated body composition data). The results of this study highlight the complex interactions between RFI, greenhouse gas, and body composition traits. A number of novel findings have resulted from the ability to generate detailed body composition data on the animals through the use of CT scanning. Whilst some of the relationships are consistent with the literature, a number have not previously been reported, or are inconsistent with the literature. This is particularly true for the relationship between body composition traits and RFI, which in cattle is assumed to be an unfavorable relationship, but in this study has been shown to be favorable. That said, basic biological understanding of the attributes of lean and adipose do support the findings of this study including that different adipose depots will likely have different relationships with different traits. Given the underlying relationships reported in this study it highlights the importance of extensive phenotyping when investigating novel traits to understand correlated responses, and that relationships will vary depending on how different traits are expressed for example, the different relationship observed between RFI and CH_4_ versus CH_4_/(CH_4_ + CO_2_). However, when armed with the knowledge that the genetic correlation between RFI and CH_4_/(CH_4_ + CO_2_) is only moderate, but both are moderately heritable traits, the use of selection indexes can ensure simultaneous genetic improvement if all traits in the index are regularly measured.

## 5 Conclusion

This study presents a comprehensive investigation into genetic parameters for RFI, CH_4,_ CO_2,_ and body composition including their phenotypic and genetic correlations for New Zealand maternal sheep genetics, as estimated from data collected in a feed intake facility. All traits investigated exhibited moderate to very high heritabilities, indicating genetic variation exists for these traits. There are several phenotypic and genetic correlations, that are different zero, some of which are novel in the literature and highlight that correlated changes in traits that affect production systems, such as body composition, can occur unless all traits are measured. We have demonstrated that selection and breeding for improved RFI and reduced CH_4_ is possible independently, but our results do suggest that residual feed intake and CH_4_/(CH_4_ + CO_2_) may be moderately negatively correlated with each other and that there is a relationship between selecting for these traits and body composition traits. It is appropriate to account for these correlations using selection index approach to ensure no unintended consequences of genetic selection on individual traits.

## Data Availability

The data supporting the results of this article are included within the article. The raw data cannot be made available, as it is property of the sheep producers in New Zealand and this information is commercially sensitive. Selected anonymized data can be made available on reasonable request to the corresponding author.

## References

[B1] Archer J. A. Proctor L. E. Byrne T. J. (2017). Economic value of selection for residual feed intake in the New Zealand sheep industry. Proc. Ass. Adv. Ani. Breed. Gene. 22, 409-412.

[B2] Archer J. Arthur P. Herd R. Parnell P. Pitchford W. (1997). Optimum postweaning test for measurement of growth rate, feed intake, and feed efficiency in British breed cattle. J. Anim. Sci. 75, 2024–2032. 10.2527/1997.7582024x 9263047

[B3] Arthur P. F. Archer J. A. Johnston D. J. Herd R. M. Richardson E. C. Parnell P. F. (2001). Genetic and phenotypic variance and covariance components for feed intake, feed efficiency, and other postweaning traits in Angus cattle. J. Anim. Sci. 79, 2805–2811. 10.2527/2001.79112805x 11768108

[B4] Bain W. E. Bezuidenhout L. Jopson N. B. Pinares-Patiño C. S. McEwan J. C. (2014). Rumen differences between sheep identified as being low or high methane emitters. Proc. Assoc. Advmt. Anim. Breed. Genet. 20, 376–378.

[B5] Ball A. Oddy V. Thompson J. (1997). “Nutritional manipulation of body composition and efficiency in ruminants,” in Recent advances in animal nutrition in Australia (Armidale, Australia: University of New England), 192–208.

[B6] Basarab J. Colazo M. Ambrose D. Novak S. McCartney D. Baron V. (2011). Residual feed intake adjusted for backfat thickness and feeding frequency is independent of fertility in beef heifers. Can. J. Anim. Sci. 91, 573–584. 10.4141/cjas2011-010

[B7] Berg R. T. Butterfield R. M. (1976). New concepts of cattle growth. Hemel Hempstead, England: Sydney University Press, Prentice/Hall Int.

[B8] Bergman E. N. (1990). Energy contributions of volatile fatty acids from the gastrointestinal tract in various species. Physiol. Rev. 70, 567–590. 10.1152/physrev.1990.70.2.567 2181501

[B9] Berry D. Crowley J. (2013). CELL BIOLOGY SYMPOSIUM, Genetics of feed efficiency in dairy and beef cattle. J. Anim. Sci. 91, 1594–1613. 10.2527/jas.2012-5862 23345557

[B10] Blaxter K. L. Claperton J. L. (1964). Prediction of the amount of methane produced by ruminants. Br. J. Nutr. 19, 511–522. 10.1079/bjn19650046 5852118

[B11] Cammack K. M. Leymaster K. A. Jenkins T. G. Nielsen M. K. (2005). Estimates of genetic parameters for feed intake, feeding behavior, and daily gain in composite ram lambs. J. Anim. Sci. 83, 777–785. 10.2527/2005.834777x 15753331

[B12] Cockrum R. Stobart R. Lake S. Cammack K. (2013). Phenotypic variation in residual feed intake and performance traits in rams. Small Rumin. Res. 113, 313–322. 10.1016/j.smallrumres.2013.05.001

[B13] Donoghue K. A. Arthur P. F. Wilkins J. F. Herd R. M. (2011). Onset of puberty and early-life reproduction in Angus females divergently selected for post-weaning residual feed intake. Anim. Prod. Sci. 51, 183. 10.1071/AN10097

[B14] Elmes S. N. Bain W. E. Greer G. J. Hickey S. M. Young E. A. Pickering N. K. (2014). BRIEF COMMUNICATION, an exploratory investigation of the effects of selection for divergence in methane emissions on rumen digesta and carcass traits in 8-month old sheep. Proc. N. Z. Soc. Ani. Prod. 74, 142–144.

[B15] Fogarty N. M. Lee G. J. Ingham V. M. Gaunt G. M. Cummins L. J. (2006). Variation in feed intake of grazing crossbred ewes and genetic correlations with production traits. Aust. J. Agric. Res. 57, 1037. 10.1071/AR05403

[B16] Fullerton G. D. Zagzebski J. A. (1980). Medical physics of CT and ultrasound: tissue imaging and characterisation. Medical physics monograph, 6. New York, NY: American Institue of Physics.

[B17] Gilmour A. R. Gogel B. Cullis B. Thompson R. (2009). ASReml user guide release 3.0. Hemel Hempstead, UK: VSN International Ltd.

[B18] Goopy J. Woodgate R. Donaldson A. Robinson D. Hegarty R. (2011). Validation of a short-term methane measurement using portable static chambers to estimate daily methane production in sheep. Anim. Feed Sci. Technol. 166-167, 219–226. 10.1016/j.anifeedsci.2011.04.012

[B19] Goopy J. P. Donaldson A. Hegarty R. Vercoe P. E. Haynes F. Barnett M. (2014). Low-methane yield sheep have smaller rumens and shorter rumen retention time. Br. J. Nutr. 111, 578–585. 10.1017/S0007114513002936 24103253

[B20] Goopy J. Robinson D. Woodgate R. Donaldson B A. Oddy B V. Vercoe P. (2016). Estimates of repeatability and heritability of methane production in sheep using portable accumulation chambers. Anim. Prod. Sci. 56, 116. 10.1071/AN13370

[B21] Gundersen H. J. G. Bendtsen T. F. Korbo L. Marcussen N. Møller A. Nielsen K. (1988). Some new, simple and efficient stereological methods and their use in pathological research and diagnosis. Acta Path. Micro. Imm. Scan. 96, 379–394. 10.1111/j.1699-0463.1988.tb05320.x 3288247

[B22] Hebart M. L. Accioly J. M. Copping K. J. Deland M. P. B. Herd R. M. Jones F. M. (2016). Divergent breeding values for fatness or residual feed intake in Angus cattle. 5. Cow genotype affects feed efficiency and maternal productivity. Anim. Prod. Sci. 58, 80. 10.1071/AN14034

[B23] Herd R. M. Velazco J. I. Smith H. Arthur P. F. Hine B. Oddy H. (2019). Genetic variation in residual feed intake is associated with body composition, behavior, rumen, heat production, hematology, and immune competence traits in Angus cattle1. J. Anim. Sci. 97, 2202–2219. 10.1093/jas/skz077 30789654 PMC6488334

[B24] Johnson P. L. Miller S. P. Knowler K. J. Bryson B. Dodds K. G. (2015). BRIEF COMMUNICATION, Modelling liveweight change to inform a residual feed intake model in sheep. Proc. N. Z. Soc. Ani. Prod. 75, 225–227.

[B25] Johnson P. L. Miller S. P. Knowler K. J. (2016). Preliminary investigations into the trait of residual feed intake in sheep. Proc. N. Z. Soc. Ani. Prod. 75, 34–37.

[B26] Johnson P. L. Wing J. Knowler K. J. Johnstone P. (2017). Investigating variation in the test length required to estimate the trait of residual feed intake in growing maternal lambs. Proc. Ass. Adv. Ani. Breed. Gene. 22, 325-328.

[B27] Johnson P. L. Knowler K. J. Wing J. Hickey S. Johnstone P. (2018). Preliminary estimates of genetic parameters for residual feed intake in sheep. Proc. World Con. Gene. App.Live. Prod. 11, 608.

[B28] Jonker A. Hickey S. Rowe S. Janssen P. Shackell G. Elmes S. (2018). Genetic parameters of methane emissions determined using portable accumulation chambers in lambs and ewes grazing pasture and genetic correlations with emissions determined in respiration chambers. J. Anim. Sci. 96, 3031–3042. 10.1093/jas/sky187 29741677 PMC6095386

[B29] Jonker A. Hickey S. M. Boma P. Woyimo Woju C. Sandoval E. MacLean S. (2020a). Individual-level correlations of rumen volatile fatty acids with enteric methane emissions for ranking methane yield in sheep fed fresh pasture. Anim. Prod. Sci. 61, 300. 10.1071/AN20128

[B30] Jonker A. Hickey S. M. McEwan J. C. Waghorn G. (2020b). “Chapter 6: Portable accumulation chambers for enteric methane determination in sheep,” in Guidelines for estimating methane emissions from individual ruminants using: GreenFeed, 'sniffers', hand-held laser detector and portable accumulation chambers (Wellington, New Zealand: Ministry for Primary Industries). Available at: https://www.mpi.govt.nz/resources-and-forms/publications/

[B31] Jopson N. B. Kolstad K. Sehested E. Vangen O. (1995). Computed tomography as an accurate and cost effective alternative to carcass dissection. Proc. Ass. Adv. Ani. Breed. Gene. 11, 635–638.

[B32] Jopson N. B. Thompson N. M. Fennessy P. F. (1997). Tissue mobilization rates in male fallow deer (*Dama danta*) as determined by computed tomography: the effects of natural and enforced food restriction. Anim. Sci. 65, 311–320. 10.1017/S1357729800016635

[B33] Kenyon P. R. Maloney S. K. Blache D. (2014). Review of sheep body condition score in relation to production characteristics. N. Z. J. Agric. Res. 57, 38–64. 10.1080/00288233.2013.857698

[B34] Koch R. Swiger L. Chambers D. Gregory K. (1963). Efficiency of feed use in beef cattle. J. Ani. Sci. 22, 486–494. 10.2527/jas1963.222486x

[B35] Lambe N. Young M. McLean K. Conington J. Simm G. (2003). Prediction of total body tissue weights in Scottish Blackface ewes using computed tomography scanning. Anim. Sci. 76, 191–197. 10.1017/S1357729800053443

[B36] Lambe N. Brotherstone S. Young M. Conington J. Simm G. (2005). Genetic relationships between seasonal tissue levels in Scottish Blackface ewes and lamb growth traits. Anim. Sci. 81, 11–21. 10.1079/ASC41670011

[B37] McClelland T. Russel A. (1972). The distribution of body fat in Scottish Blackface and Finnish Landrace lambs. Anim. Sci. 15, 301–306. 10.1017/s0003356100011569

[B38] McLean N. J. Jopson N. B. Campbell A. W. Knowler K. Behrent M. Cruickshank G. (2006). An evaluation of sheep meat genetics in New Zealand, the central progeny test (cpt). Proc. N. Z. Soc. Ani. Prod. 66, 368–372.

[B39] MFE (2017). New Zealand’s greenhouse gas inventory 1990-2017. Available at: https://www.mfe.govt.nz/node/23304/ (Accessed 04 01, 2019).

[B40] Muir S. K. Linden N. Knight M. Behrendt R. Kearney G. (2018). Sheep residual feed intake and feeding behaviour, are 'nibblers' or 'binge eaters' more efficient? Anim. Prod. Sci. 58, 1459. 10.1071/an17770

[B41] Paganoni B. Rose G. Macleay C. Jones C. Brown D. J. Kearney G. (2017). More feed efficient sheep produce less methane and carbon dioxide when eating high-quality pellets. J. Anim. Sci. 95, 3839–3850. 10.2527/jas2017.1499 28992015

[B42] Palsson H. (1939). Meat qualities in the sheep with special reference to Scottish breeds and crosses. I. J. Agric. Sci. 29, 544–626. 10.1017/S0021859600052242

[B43] Pickering N. de Haas Y. Basarab J. Cammack K. Hayes B. Hegarty R. (2013). Consensus methods for breeding low methane emitting animals. A White Paper prepared by the Animal Selection, genetics and genomics network of the livestock research group of global research alliance for reducing greenhouse gases from agriculture. https://irp.cdn-website.com/0978b13c/files/uploaded/MPWG_whitepaper.pdf.

[B44] Pinares-Patiño C. S. Hickey S. M. Young E. A. Dodds K. G. MacLean S. Molano G. (2013). Heritability estimates of methane emissions from sheep. Animal 7, 316–321. 10.1017/S1751731113000864 PMC3691003 23739473

[B45] Redden R. Surber L. Grove A. Kott R. (2013). Growth efficiency of Ewe lambs classified into residual feed intake groups and pen fed a restricted amount of feed. Small Rumin. Res. 114, 214–219. 10.1016/j.smallrumres.2013.07.002

[B46] Robinson D. L. Goopy J. P. Hegarty R. S. Oddy V. H. Thompson A. N. Toovey A. F. (2014). Genetic and environmental variation in methane emissions of sheep at pasture. J. Anim. Sci. 92, 4349–4363. 10.2527/jas.2014-8042 25149329

[B47] Rowland K. Ashwell C. M. Persia M. E. Rothschild M. F. Schmidt C. Lamont S. J. (2019). Genetic analysis of production, physiological, and egg quality traits in heat-challenged commercial white egg-laying hens using 600k SNP array data. Genet. Sel. Evol. 51, 31. 10.1186/s12711-019-0474-6 31238874 PMC6593552

[B48] Tortereau F. Marie-Etancelin C. Weisbecker J. L. Marcon D. Bouvier F. Moreno-Romieux C. (2020). Genetic parameters for feed efficiency in Romane rams and responses to single-generation selection. Animal 14, 681–687. 10.1017/S1751731119002544 31640830

[B49] Walkom S. F. Brien F. D. Hebart M. L. Pitchford W. S. (2015). The impact of selecting for increased Ewe fat level on reproduction and its potential to reduce supplementary feeding in a commercial composite flock. Anim. Prod. Sci. 56, 698. 10.1071/AN14579

[B50] Williams Y. J. Pryce J. E. Grainger C. Wales W. J. Linden N. Porker M. (2011). Variation in residual feed intake in Holstein-Friesian dairy heifers in southern Australia. J. Dairy Sci. 94, 4715–4725. 10.3168/jds.2010-4015 21854946

[B51] Wright I. Russel A. (1984). Partition of fat, body composition and body condition score in mature cows. Anim. Sci. 38, 23–32. 10.1017/S0003356100041313

[B52] Young M. J. Nsoso S. J. Logan C. M. Beatson P. R. (1996). Prediction of carcass tissue weight *in vivo* using live weight, ultrasound or x-ray ct measurements. Proc. N. Z. Soc. Ani. Prod. 56, 205–211.

